# *Mycoplasma agalactiae* MAG_5040 is a Mg^2+^-Dependent, Sugar-Nonspecific SNase Recognised by the Host Humoral Response during Natural Infection

**DOI:** 10.1371/journal.pone.0057775

**Published:** 2013-02-28

**Authors:** Carla Cacciotto, Maria Filippa Addis, Elisabetta Coradduzza, Laura Carcangiu, Anna Maria Nuvoli, Gessica Tore, Gian Mario Dore, Daniela Pagnozzi, Sergio Uzzau, Bernardo Chessa, Marco Pittau, Alberto Alberti

**Affiliations:** 1 Dipartimento di Medicina Veterinaria, Università degli Studi di Sassari, Sassari, Italy; 2 Porto Conte Ricerche Srl, Tramariglio, Alghero (SS), Italy; 3 Dipartimento di Scienze Biomediche, Università degli Studi di Sassari, Sassari, Italy; Miami University, United States of America

## Abstract

In this study the enzymatic activity of *Mycoplasma agalactiae* MAG_5040, a magnesium-dependent nuclease homologue to the staphylococcal SNase was characterized and its antigenicity during natural infections was established. A UGA corrected version of MAG_5040, lacking the region encoding the signal peptide, was expressed in *Escherichia coli* as a GST fusion protein. Recombinant GST-MAG_5040 exhibits nuclease activity similar to typical sugar-nonspecific endo- and exonucleases, with DNA as the preferred substrate and optimal activity in the presence of 20 mM MgCl_2_ at temperatures ranging from 37 to 45°C. According to *in silico* analyses, the position of the gene encoding MAG_5040 is consistently located upstream an ABC transporter, in most sequenced mycoplasmas belonging to the *Mycoplasma hominis* group. In *M. agalactiae*, MAG_5040 is transcribed in a polycistronic RNA together with the ABC transporter components and with MAG_5030, which is predicted to be a sugar solute binding protein by 3D modeling and homology search. In a natural model of sheep and goats infection, anti-MAG_5040 antibodies were detected up to 9 months post infection. Taking into account its enzymatic activity, MAG_5040 could play a key role in *Mycoplasma agalactiae* survival into the host, contributing to host pathogenicity. The identification of MAG_5040 opens new perspectives for the development of suitable tools for the control of contagious agalactia in small ruminants.

## Introduction

Mycoplasmas are the smallest and simplest self-replicating prokaryotes. They evolved from Gram-positive bacteria following a regressive process that led to the reduction of genomic resources to an essential minimum [1]. As a consequence of their extremely limited biosynthetic capabilities, mycoplasmas adapted to a parasitic lifestyle, relying on the host for many metabolic precursors [1]. In particular, mycoplasmas lost the ability to synthesize *de novo* purine and pyrimidine bases, and dramatically depend on the salvage pathway [2], [3], [4] for producing nucleotide precursors. Through the salvage pathway, free bases and nucleosides released from the breakdown of nucleic acids are converted back into nucleotides, which can therefore be recycled for bacterial nucleic acids production and for DNA repair. A possible exception to this is *Mycoplasma penetrans* (*M. penetrans*), which lacks the uridine kinase gene but has a uracil phosphoribosyl transferase and an orotate-related pathway for UMP production in pyrimidine metabolism [5]. Indeed, mycoplasmas evolutionary constraints in host adaptation forced to maintain membrane key components for extracellular degradation of nucleic acids, such as nucleases, and for transporting selected nucleotides precursors through the membrane, such as ATP-binding cassette (ABC) transport systems [1]. The importance of nucleases in mycoplasmas life cycle is reinforced by their detection in at least 20 *Mycoplasma* species [6], [7], [8], [9], [10], [11], [12], [13]. Several studies indicate that many of these enzymes are implicated in host pathogenicity and cytotoxicity. As an example, the Staphylococcal Nuclease (SNase) homologue of *M. hyorhinis* is able to induce chromatin internucleosomal degradation and apoptotic changes in epithelial cells [11]. Similarly, it was demonstrated that the endonuclease P40 of *M. penetrans* triggers apoptosis in lymphocytes *in vitro* [14]. In a previous study, we systematically characterized the liposoluble proteome of *M. agalactiae* PG2^T^ [15]. Among the identified proteins, we demonstrated the expression of MAG_5040, which was classified, according to gene ontology analyses, as a surface lipoprotein containing the conserved SNase domain of *Staphylococcus aureus* (*S. aureus*). Proteins homologues to SNase have been identified in many bacteria, and in several mycoplasmas [6], [16], [17], [18], [19]. In this study we characterized the *in vitro* activity of the putative *M. agalactiae* SNase MAG_5040, and we investigated its expression and antigenic properties during natural infection. The gene encoding MAG_5040 was cloned and GST-tagged in an *E. coli* expression system. Substrate specificity and biochemical properties of the purified recombinant protein (rGST-MAG_5040) were examined. Recombinant cleaved MAG_5040 was also used to detect specific antibodies during different stages of infection in the natural hosts (sheep and goats), and to determine its reactivity with hyperimmune sera raised against selected mycoplasma species, as a preliminary investigation of potential SNase homologues expressed in other *Mycoplasma* species.

## Materials and Methods

### Ethics Statement

This study was approved by the ethics committee of the University of Sassari. Blood sampling and pharmacological treatment of infected animals were operated by a veterinary practitioner authorized by the National Health System, after obtaining permission from the sheep owner. Animals where moved and transported by the shepherd during routine management of the flock in accordance with D.P.R. 8 Febbraio 1954, n. 320. Rabbit hyperimmune sera were kindly provided in 1996 by E.A. Freundt (Institute of Medical Microbiology, University of Aarhus, Denmark).

### *In silico* Analyses

The *M. agalactiae* MAG_5040 protein sequence (YP_001256642) was submitted to BLASTP [20], and 8 sequences representative of 5 of the 8 mycoplasma clusters of the *M. hominis* group were selected. Sequences of the *M. sualvi*, *M. lipophilum*, and *M. equigenitalium* clusters were not available, since the genomes of these mycoplasmas have not yet been sequenced. Regions flanking MAG_5040 homologs were also investigated by homology search in the 8 mycoplasmas. These analyses were extended to three additional sequences selected outside the *M. hominis* group (*M. genitalium* and *M. pulmonis*) and outside mycoplasmas (*S. aureus* subspecies *aureus*). MAG_5040 protein sequence was aligned to the homologues sequences identified in *M. bovis* (YP_006471195), *M. fermentans* (YP_004136712), *M. synoviae* (YP_278410), *M. hyorhinis* (YP_003856075), *M. hyopneumoniae* (YP_115890), *M. ovipneumoniae* (ZP_09312358), *M. pulmonis* (NP_325856), *M. hominis* (YP_003302610), *M. genitalium* (NP_072849), *M. pneumoniae* (NP_109821), and *S. aureus* (YP_001316549) by CLUSTALW [21]. Genetic distances among the operational taxonomic units (OTUs) were computed using the Equal Input method [22] and were used to construct neighbor-joining (NJ) trees [23]. Genetic distances and trees were calculated using MEGA5 [24]. MAG_5040 putative lipoprotein cleavage site and conserved domains were identified with LipoP [25] and PROSITE scan [26], respectively. MAG_5030 and MAG_5080 3D modeling and structures were investigated by using the Protein Homology/analogY Recognition Engine (Phyre) V 2.0 [27].

### Bacterial Strains and Culture Conditions

*M. agalactiae* PG2^T^ was grown in PPLO medium supplemented with 20% heat inactivated horse serum and 500 µg/ml ampicillin, at 37°C with constant agitation. Mycoplasmas were collected by centrifugation (10 min at 10,000×g at 4°C), and washed three times with ice-cold PBS. Pellets were stored at −80°C until use. *E. coli* strains were grown in Luria-Bertani broth or on Luria-Bertani agar [28].

### Cloning, Expression, and Purification of rMAG_5040

Total DNA was extracted from mycoplasma pellets with DNeasy Blood & Tissue Kit (Qiagen). In order to express MAG_5040 in fusion with glutathione-S-transferase (GST), a version of the MAG_5040 gene excluding the region encoding the signal peptide (amino acids 1 to 25) was amplified with primers MAG_5040/BamHI/F and MAG_5040/EcoRI/R (Table S1). PCR recipe and cycling conditions were set according to vendor recommendations for Platinum®Pfx DNA Polymerase (Invitrogen). The PCR product was resolved by agarose gel electrophoresis and purified with the QIAquick Gel Extraction kit (Qiagen), digested with BamHI and EcoRI, and ligated with the Rapid DNA Dephos & Ligation Kit (Roche) to a pGEX-2T vector (GE Healthcare), previously digested with the same enzymes. One Shot TOP10 Chemically Competent *E. coli* (Invitrogen) were transformed with the ligation product, and clones containing the recombinant vector (pGEX-2T/MAG_5040) were selected for ampicillin resistance. pGEX-2T/MAG_5040 was purified with the PureLink™ Quick Plasmid Miniprep Kit (Invitrogen). Automated Sanger sequencing confirmed the correct cloning of MAG_5040 sequence into the vector. To avoid the expression of truncated proteins, 7 mycoplasma TGA tryptophan codons contained in MAG_5040 were mutagenized to TGG by using the QuikChange® Site-Directed Mutagenesis Kit (Stratagene), following manufacturer’s instructions. Primers used for mutagenesis (MAG_5040/MUT) are summarized in Table S1. The final vector (pGEX-2T/rMAG_5040) containing the mutagenized MAG_5040 gene was extracted as described above and sequenced. *E. coli* BL21(DE3) were transformed with pGEX-2T/rMAG_5040 by means of RapidTransit™ Transformation Kit (Sigma-Aldrich) according to manufacturer’s instructions, and positive clones were selected for ampicillin and chloramphenicol resistance. Expression of rMAG_5040 was induced by adding IPTG (0.1 mM final concentration) and incubating at 30°C under constant agitation for 4 hours. The rGST-MAG_5040 fusion protein was purified by means of affinity chromatography with Glutathione Sepharose™ High Performance (GE Healthcare), and buffer-exchanged to PBS in a 30 kDa NMWL Amicon Ultra-15 centrifugal filter unit (Millipore). In order to obtain rMAG_5040, GST was cleaved from rGST-MAG_5040 by using thrombin (GE Healthcare). Both rGST-MAG_5040 and rMAG_5040 concentrations were evaluated with the BCA Protein Assay kit (Pierce).

### Nuclease Activity Assays

Approximately 4 µg of rGST-MAG_5040 were incubated at 37°C in 100 µl reaction buffer (25 mM Tris-HCl, pH 8.8, 5 mM MgCl_2_, 5 mM CaCl_2_) containing 1 to 5 µg of nucleic acid substrate. Aliquots (10 µl) were sampled at different times of incubation, and reaction was stopped in each collected tube by adding EDTA at the final concentration of 20 mM. Digestion products were analyzed by agarose gel electrophoresis and documented as described above. Exonuclease and endonuclease activities were evaluated by digesting both linear DNA (UltraPure™ Calf Thymus DNA Solution, Invitrogen) and the circular plasmid pGEX-2T/rMAG_5040. Substrate specificity was investigated with ssDNA (M13 DNA, New England Biolabs) and total RNA purified from mid-log *E. coli* cultures. Optimal reaction conditions were defined by varying calcium and magnesium concentrations, ionic strength, and temperature in triplicate digestion reactions of the plasmid pGEX-2T/rMAG_5040. In order to examine the nuclease activity of rGST-MAG_5040 in the absence of exogenously supplied divalent cations, EDTA was added to the reaction (5 mM final concentration). In order to rule out carryover of *E. coli* nucleases along with the fusion protein, GST was expressed in *E. coli*, purified, and used as a negative control in all assays.

### Longitudinal Study and Sera Collection

Ten sheep were selected from a Contagious Agalactia (CA) free flock in North Sardinia (Italy). In order to confirm the absence of *M. agalactiae*, sheep (group A) were tested by milk mycoplasma isolation in PPLO [29], by *M. agalactiae* rP48 ELISA [30], and by western blotting against total protein extract of *M. agalactiae* PG2^T^. Five sheep belonging to group A and negative to the three tests were moved into an infected flock (group B), and placed in close contact with symptomatic animals actively shedding *M. agalactiae* in milk. Sera were collected at time of arrival (T_0_) from the 5 negative group A sheep, and from both positive (n = 5) and negative (n = 5) animals of the group B. Sera sampling was repeated every other week for a total of 36 weeks (9 months). In order to confirm infection of negative sheep during the longitudinal study, animals were tested both serologically and culturally as described above, at each sampling occasion. Sera obtained from both positive and negative group B sheep where pooled and used as positive and negative controls, respectively.

Also, a panel of 10 well-characterized sera of an outbreak of CA occurred in Sicily were kindly provided by the ”Istituto Zooprofilattico della Sardegna”. Furthermore, 16 high titer sera were obtained from the Department of Veterinary Science (University of Turin) and came from 8 naturally infected goats sampled at two weeks distance in Piedmont (continental Northern Italy). Nine rabbit hyperimmune sera raised against whole cell preparations of eight mycoplasma species, namely *M. agalactiae* PG2^T^, *M. mycoides* subsp. *capri* PG3, *M. capricolum* subsp. *capricolum* CK, *M. arginini* G230, *M. canadense* C275, *M. ovipneumoniae* Y98, *M. putrefaciens* KS1, *M. mycoides* subsp. *capri* LC, and *M. capricolum* subsp. *capripneumoniae* were also used in this study.

### Western Immunoblotting

For SDS-PAGE, approximately 200 ng of cleaved rMAG_5040 or alternatively total protein extracts of *M. agalactiae* PG2^T^ were run in single well 10% polyacrylamide gels (corresponding to 6–10 ng/lane), and transferred into nitrocellulose membranes with a Mini-Trans-Blot Cell (Bio-Rad), at 250 mA for one hour. After blotting, membranes were blocked with PBS-0.05% Tween-20 (PBS-T) containing 5% skim milk, and then incubated for one hour in a Multiscreen apparatus (Bio-Rad) alternatively with sera obtained from *M. agalactiae* naturally infected sheep and goats of geographically distant Italian Regions (Sardinia, Sicily, and Piedmont), or with rabbit hyperimmune sera raised against different *Mycoplasma* species. After incubation with primary antibodies, membranes were washed five times with PBS-T and incubated with the appropriate HRP-conjugated secondary antibodies (Southern Biotech). After five washes, membranes were developed with Chemiluminescent Peroxidase Substrate (Sigma-Aldrich) and images were acquired with a VersaDoc MP 4000 Imaging System (Bio-Rad).

## Results

### *In silico* Analysis

**Figure 1:**
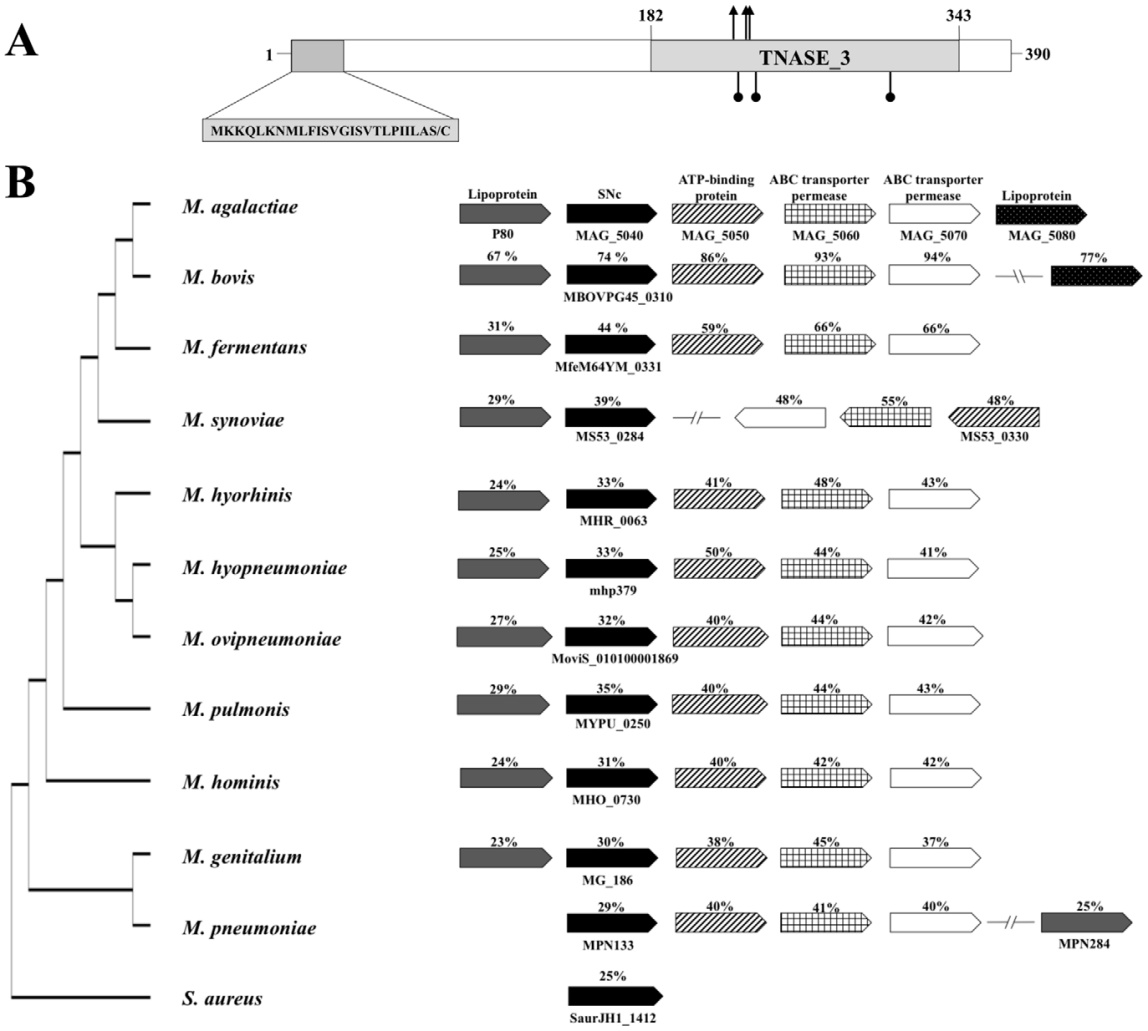
MAG_5040 organization and position of homologs in the genome of phylogenetically related bacteria. (A) MAG_5040 schematic diagram. MAG_5040 is comprised of 390 amino acids and contains a hydrophobic N-terminal signal sequence and a prokaryotic lipoprotein cleavage site (indicated by the grey box). Amino acids 182 through 343 have significant identity and similarity with the TNASE_3 domain profile of the *Staphylococcus aureus* thermonuclease (SNc). Conserved amino acid residues involved in binding of divalent ions are indicated by arrowed vertical lines. Position of amino acids comprising the active catalytic site are shown as vertical lines with round tips. (B) Physical map of mycoplasma homologs of the putative MAG_5030-MAG_5040-MAG_5050-MAG_5060-MAG_5070 ABC transport system. Homologous proteins are shaded in the same pattern, and their amino acid identities to the corresponding *M. agalactiae* PG2^T^ proteins are indicated above each row. Phylogeny based on the amino acid alignment of the SNc homologs is shown on the left.

**Figure 2:**
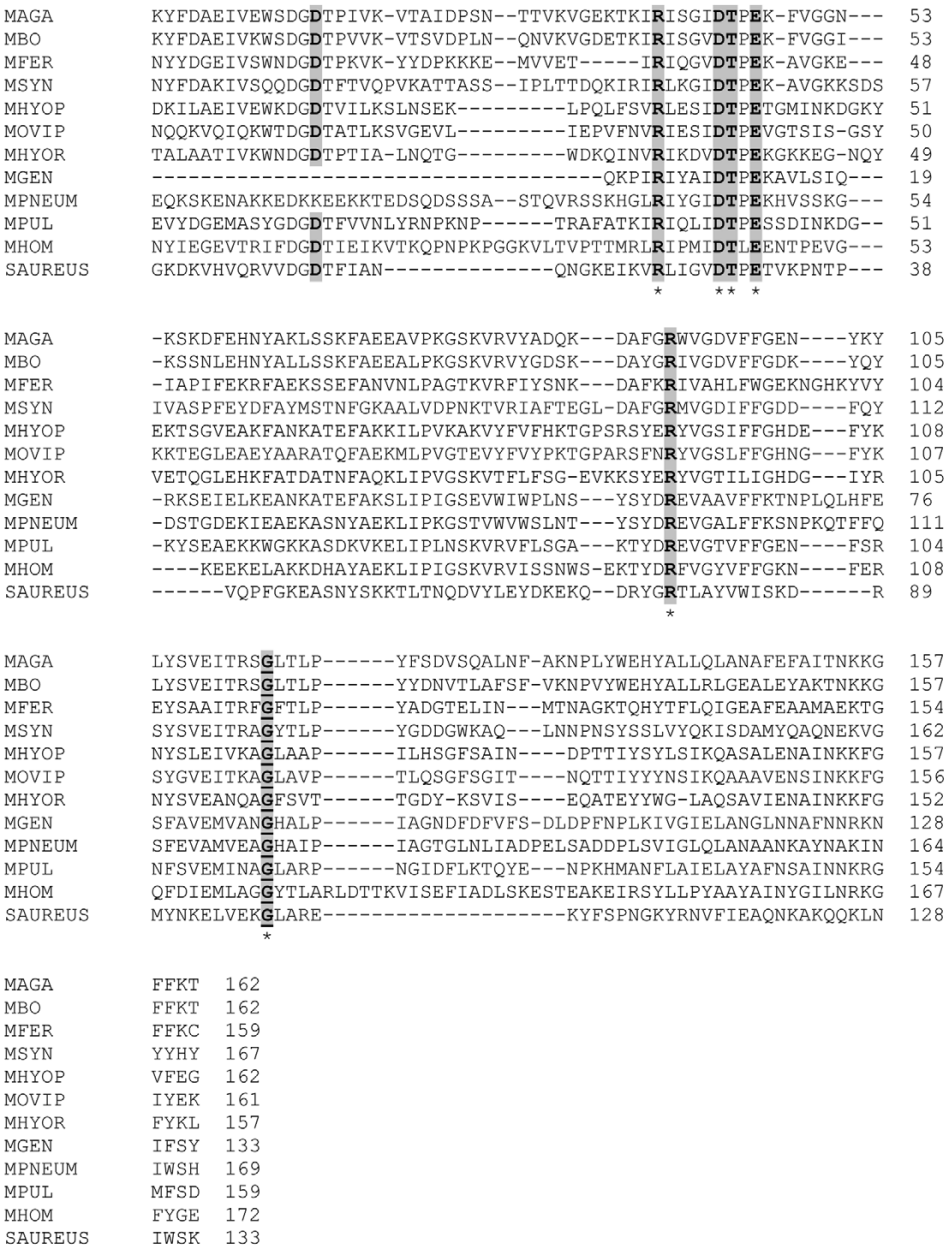
Multiple sequence alignment of the TNASE_3 domain of *M. agalactiae* PG2^T^ MAG_5040 with homologous proteins identified in other mycoplasma species, and with the SNc of *Staphylococcus aures.* Positions are numbered according to the first amino acid of this domain in each bacterial species. Conserved amino acid residues involved in cations binding and encompassing the catalytic site are shown in boldface. An additionally conserved glycine residue is underlined and in boldface. Asterisks indicate positions conserved in all the bacteria aligned.

The MAG_5040 gene encodes a putative protein of 390 amino acid residues with a predicted molecular mass of 44.67 kDa and a *pI* of about 8.5. LipoP analysis classifies MAG_5040 as a putative lipoprotein, with a classical signal peptide of 25 amino acids and a typical cysteine cleavage site at residue 25 (Figure 1A). PROSITE scan of MAG_5040 reveals the presence of the nuclease domain TNASE_3, characteristic of the *S. aureus* thermonuclease [16], [17], [18], [19]. The TNASE_3 domain (Figure 1A, Figure 2) spans positions 182–343, and contains two aspartates and a threonine located at positions 195, 225, and 226, respectively. These amino acids are conserved among mycoplasmas of the *M. hominis* group, and are involved in the binding of divalent ions. The amino acid residues arginine, glutamate, and arginine, respectively located in the TNASE_3 motive at positions 220, 228, and 273 are also strictly conserved and comprise the active catalytic site. Additionally, a glycine residue is conserved at position 296. Figure 1B shows the alignment of MAG_5040 gene and its flanking regions with the corresponding regions of 10 selected *Mycoplasma* species and with *S. aureus* subspecies *aureus*. In *M. agalactiae*, MAG_5040 is located immediately upstream of 3 genes encoding an ABC transport system. MAG_5050, the predicted ATP-binding protein, contains the two ATP-binding domains that couple ATP hydrolysis to transport. The two genes MAG_5060 and MAG_5070 encode the transmembrane permeases that are associated with the import of the substrate. MAG_5050, MAG_5060, MAG_5070 shows similarity to putative sugar ABC transporter components of mycoplasma species belonging to the hominis group (Figure 1B), and of other distant bacteria. Indeed BLASTP sequence comparisons identify homologues of MAG_5050, MAG_5060, MAG_5070 in *M. crocodyli*, *M. pneumoniae*, *M. gallisepticum*, *Achromobacter* spp., *Roseobacter* sp., *Clostridium* sp., *Octadecabacter antarcticus*, *Lactobacillus* spp., and many other bacterial genera (data not shown). Similarly, MAG_5040 homologues are present in about 60 Gram-negative and Gram-positive bacterial species. With one exception (*M. synoviae*, Figure 1B), the sequential distribution of MAG_5040 and of the ABC transporter genes is highly conserved in mycoplasmas belonging to the *M. hominis* group (Figure 1B). In addition, position of MAG_5030 upstream of the putative SNase is also conserved, at least in the same mycoplasmas. *Mycoplasma agalactiae* MAG_5030 encodes the antigenic lipoprotein P80 [31]. On the contrary MAG_5080, located downstream of the ABC transporter, shows significant homology uniquely with the MBOVPG45_0305 gene of *M. bovis*, also positioned in this mycoplasma downstream of the ABC transporter permeases, but with the interposition of the small open reading frame MBOVPG45_0306 (Figure 1B).

In 3D modeling and structure prevision analyses conducted with Phyre^2^, P80 matches the periplasmic binding protein-like II superfamily, with 99.6% confidence (based on 55% of the MAG_5030 sequence). Significant values of confidence >90% are also observed with families of solute binding proteins mostly associated with sugar transport (Figure S1). On the contrary, in similar analyses MAG_5080 does not generate any significant structure prevision (confidence of the highest scoring template <50%).

### Cloning, Expression, and Purification of rMAG_5040

The MAG_5040 gene, lacking the region encoding the signal peptide, was successfully amplified and cloned into the expression vector pGEX-2T. In order to avoid the expression of truncated versions of the recombinant protein in *E. coli*, seven TGA tryptophan codons were successfully changed into TGG by site directed mutagenesis, as confirmed by sequencing. Upon induction with IPTG, SDS-PAGE analysis of *E. coli* BL21(DE3) cells transformed with pGEX-2T/rMAG_5040 revealed the overexpression of a soluble protein of about 68 kDa, according to the predicted rGST-MAG_5040 molecular mass. This band was absent in uninduced *E. coli* cells (Figure S2). The recombinant protein rGST-MAG_5040 was successfully purified by means of affinity chromatography. Thrombin cleavage of rGST-MAG_5040 produced two bands of about 26 and 42 kDa in SDS-PAGE, corresponding in size to the GST fusion partner and to rMAG_5040, respectively (Figure S2).

### Nuclease Activity of rMAG_5040

**Figure 3:**
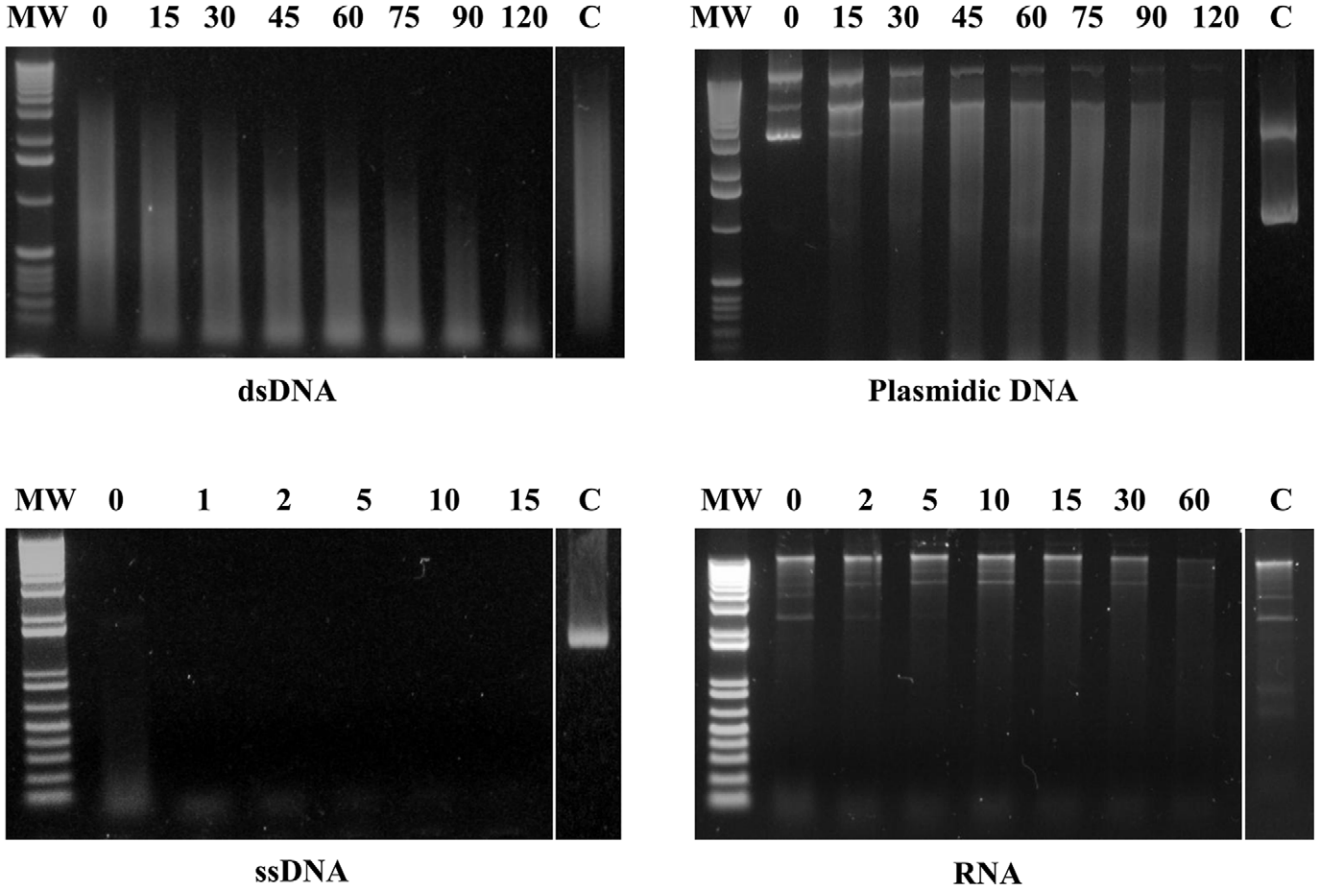
Nuclease activity and substrate specificity of recombinant GST-MAG_5040. Approximately 4 µg of rGST-MAG_5040 were incubated with calf thymus dsDNA (top left panel), closed circular plasmid DNA (pGEX-2T/rMAG_5040, top right panel), phage M13 ssDNA (bottom left panel), or *E. coli* total RNA (bottom right panel). An aliquot of each reaction mixture was analysed at different times, as indicated for each lane. C indicates endpoint reaction of the negative undigested control (GST only). Both 1 Kb (upper panels) and 1 Kb Plus (bottom panels) DNA ladders were used (Invitrogen) and indicated as MW in far left lanes of each panel.

**Figure 4:**
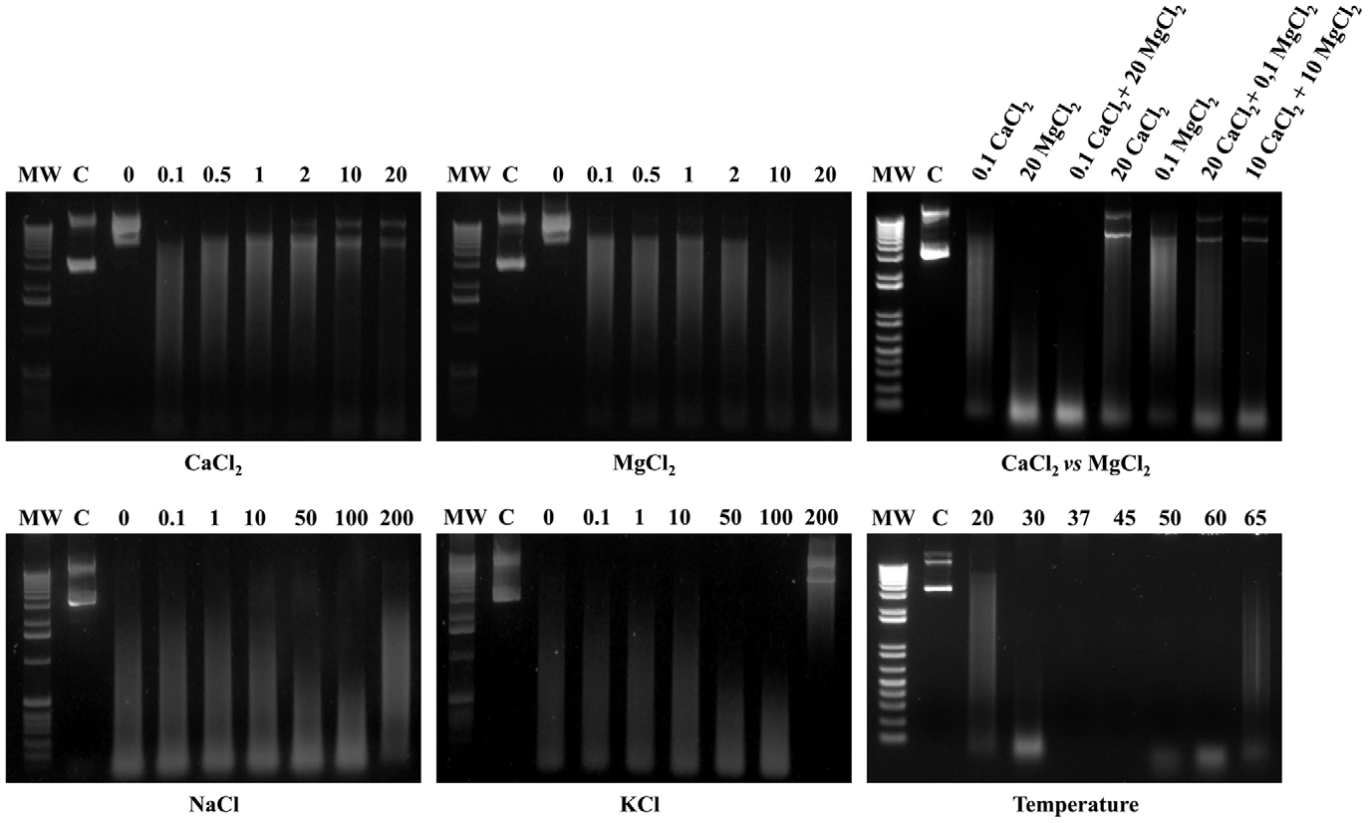
Effects of Ca^2+^, Mg^2+^, combined Ca^2+^+Mg^2+^, ionic strength, and temperature on MAG_5040 nuclease activity. The nuclease activity and stability under different tested conditions were evaluated by loading on a 1% agarose gel approximately 10 µl of each of the endpoint reactions. The far left lane of each panel was loaded with the molecular weight marker (MW). Both 1 Kb (left and central panels) and 1 Kb Plus (top and bottom right panels) DNA ladders were used (Invitrogen). In each panel, lanes designated C were loaded with untreated plasmid DNA in agarose loading buffer. Concentrations expressed in mM and temperatures in °C are indicated in the appropriate panels **(**Ca^2+^, top left panel; Mg^2+^, top middle panel; combined Ca^2+^+Mg^2+^, top right panel; ionic strength, bottom left and middle panels; and temperature, bottom right panel).

In order to evaluate MAG_5040 nuclease activity, rGST-MAG_5040 was incubated with different nucleic acid substrates under different conditions. Since it has been demonstrated that most *Mycoplasma* nucleases show their maximum activity when Ca^2+^ and Mg^2+^ ions are combined in the reaction buffer [10], substrate specificity of MAG_5040 was initially evaluated in the presence of both 5 mM CaCl_2_ and 5 mM MgCl_2_. When linear dsDNA (calf thymus DNA) was incubated with GST-rMAG_5040, the amount of intact dsDNA decreased during incubation, and that was associated with the appearance of a smear in the gel, whose average size became lower over time (Figure 3, upper left panel), indicating an exonuclease activity of the protein. Similarly, endonuclease activity was assessed by using a dsDNA circular plasmid (pGEX-2T/rMAG_5040) as a substrate (Figure 3, upper right panel). Recombinant GST-MAG_5040 appeared to be far more active against ssDNA (M13 DNA) than against dsDNA. Indeed, high degree of ssDNA degradation could be instantly observed at T_0_. After 1 minute, M13 DNA was almost totally digested (Figure 3, lower left panel). Recombinant GST-MAG_5040 was also found to be active, although to a lesser extent, against total RNA extracted from *E. coli* (Figure 3, lower right panel). Digestions of the different substrate were always coupled with adequately matched negative controls (Figure 3). Optimal biochemical conditions were investigated and defined by evaluating the degree of degradation of plasmid DNA in the presence of increasing concentrations of divalent cations (Ca^2+^ and Mg^2+^), and by varying ionic strength (Na^+^ and K^+^), and temperature (Figure 4). Recombinant GST-MAG_5040 did not show any nuclease activity when reaction buffers were supplemented with EDTA (5 mM final concentration), confirming the biochemical requirement for divalent ions (data not shown). When reaction buffer was supplemented with increasing concentrations of MgCl_2,_ a corresponding increase of the ability to digest circular dsDNA was also observed, with apparent maximum efficiency of rGST-MAG_5040 in the presence of 20 mM MgCl_2_ (Figure 4, top middle panel). On the contrary when MgCl_2_ was replaced with CaCl_2_ in similar experiments, a progressive inhibitory action of Ca^2+^ on the activity of rGST-MAG_5040 could be surprisingly observed (Figure 4, top left panel). Thus, with dsDNA as substrate, MAG_5040 shows a strong preference for Mg^2+^ over calcium ions. The efficiencies of dsDNA digestions conducted by combining 20 mM MgCl_2_ to 0.1 mM CaCl_2_ and by using 20 mM MgCl_2_ alone were comparable (Figure 4, top right panel).

The influence of ionic strength on rGST-MAG_5040 activity was investigated by varying NaCl and KCl concentration (Figure 4, lower left and middle panels). We found that increasing either NaCl or KCl salt concentrations up to 100 mM enhanced the rGST-MAG_5040 activity. On the contrary, at 200 mM of any of the two salts nuclease activity was inhibited, especially with KCl. Recombinant GST-MAG_5040 performed best in a range of temperatures between 37°C and 45°C, while at 65°C and at 20°C reaction was appreciatively inhibited (Figure 4, lower right panel).

### Antigenic Properties of rMAG_5040

**Figure 5:**
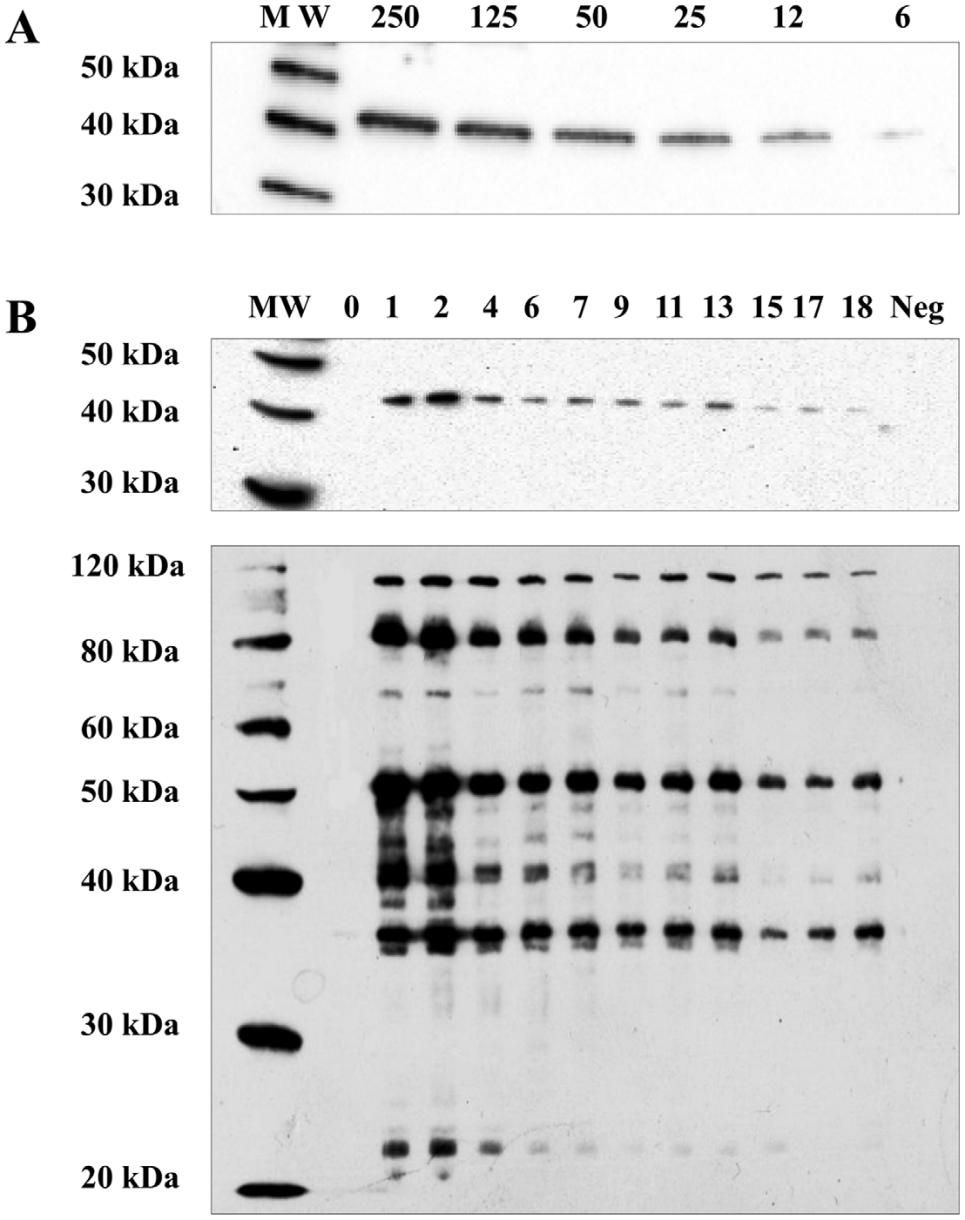
MAG_5040 antigenic properties. (A) Sensitivity of western blotting based on recombinant MAG_5040. Decreasing amounts of cleaved rMAG_5040 (250 to 6 ng) were run in a 10% polyacrylamide gel and tested against a pool of high titre *M. agalactiae* naturally infected sheep sera. (B) Reactivity of rMAG_5040 (upper panel) and total *M. agalactiae* protein lysate (bottom panel) with sera collected from a sheep selected as representative of the longitudinal study. Sera were collected every two weeks for 9 months (numbers in lanes indicates selected sampling occasions, from T_0_ to T_18_). Neg indicates a pool of negative sera used as control in western blotting. Protein ladders (Magic Marker XP, Invitrogen) were loaded in lanes designated MW.

A previous study suggested that MAG_5040 is a surface lipoprotein expressed in cultured *M. agalactiae* PG2^T^ [15]. In order to investigate the antigenic properties of MAG_5040, and to evaluate its expression in naturally infected hosts, a longitudinal study was performed on sera collected from sheep at different stages of infection. In addition, the reactivity of a panel of well-characterized goat and sheep sera from two unrelated outbreaks was also evaluated. At first, the antigenicity of MAG_5040 was established, and the sensitivity of western blotting based on its recombinant form was then assessed. Figure 5A shows the reactivity of a pool of sera obtained from naturally infected sheep against decreasing amounts of the cleaved recombinant protein. A strong reactivity of the pool of sera against rMAG_5040 could be observed down to 12 ng of recombinant protein, with 6 ng of protein still associating to a weak signal. A pool of sera obtained from negative sheep tested always negative in similar experiments (data not shown).

**Figure 6:**
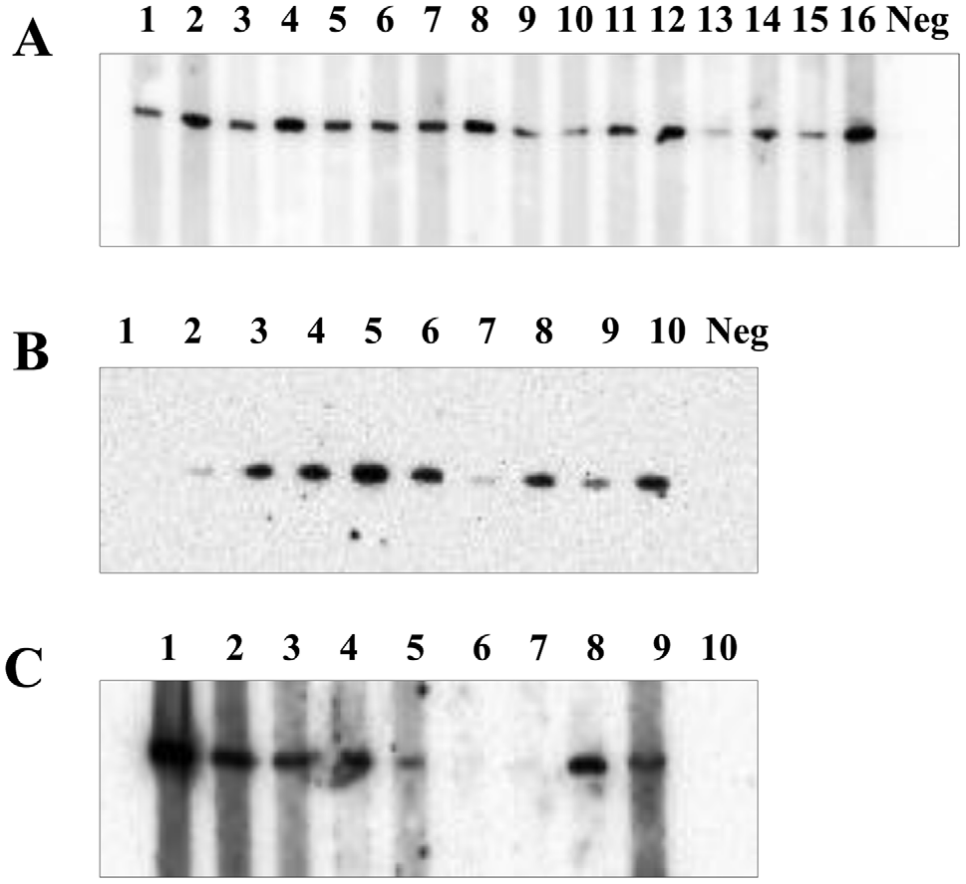
MAG_5040 western blotting reactivity with sera collected from naturally infected hosts, and indirect detection of homologs in selected mycoplasma species. 200 ng of cleaved rMAG_5040 were run in single well in 10% polyacrylamide gels and probed with sera collected alternatively from Piedmont goats naturally infected with *M. agalactiae* (A) or from sheep selected from an outbreak of contagious agalactia occurred in Sicily (B). In A, odd lanes (1 to 15) identify sera collected from 8 different goats. Even numbers (2 to 16) indicate sera taken from the same goats at two weeks distance. In B lanes 1 to 10 were probed with sera collected from 10 sheep. Neg in all panels designates lanes in which a pool of negative sera was loaded. (C) Reactivity of of cleaved rMAG_5040 (200 ng) with rabbit hyperimmune sera raised against *M. agalactiae* PG2^T^ (1), *M. mycoides* subsp. *capri* PG3 (2), *M. capricolum* subsp. *capricolum* CK (3), *M. arginini* G230 (4), *M. canadense* C275 (5), *M. ovipneumoniae* Y98 (6), *M. putrefaciens* KS1 (7), *M. mycoides* subsp. *capri* LC (8), and *M. capricolum* subsp. *capripneumoniae* (9). Lane 10 equals lane 1 except that no primary antibody was used.

In order to select a number of negative sheep from a flock with no history of CA, 10 sheep (group A) where tested by mycoplasma isolation, rP48 ELISA, and western blotting (see Material and Methods). Five group A sheep negative both serologically and culturally were moved to a mycoplasma-infected flock (group B). When rMAG_5040 was probed with sera collected from group A sheep placed in close contact with group B sheep, antibodies against MAG_5040 could be detected starting from T_1_ (2 weeks later) up to T18 (36 weeks, 9 months) in 3 out of 5 sheep. Western blotting evaluation performed on total protein lysates of *M. agalactiae* PG2^T^ against the same sera showed that the 3 group A sheep were already infected at T_1_. Mycoplasma isolation and rP48 ELISA were consistent with western blotting. Sera collected during the same sampling occasions from culturally and serologically negative sheep never reacted with rMAG_5040 (data not shown). Figure 5B summarizes the results of the longitudinal study. A strong reactivity was also observed when rMAG_5040 was tested against the two panels of well characterized sera obtained from CA outbreaks occurred in Piedmont goats and Sicilian sheep (Figure 6A and 6B).

To tentatively investigate the presence of expressed MAG_5040 homologues in selected mycoplasma species, we tested the reactivity of specific rabbit hyperimmune sera against rMAG_5040 (Figure 6B). A positive reaction was observed with specific α - *M. mycoides* subsp. *capri* PG3, *M. capricolum* subsp. *capricolum* CK, *M. arginini* G230, *M. canadense* C275, *M. mycoides* subsp. *capri* LC, *M. capricolum* subsp. *capripneumoniae*.

## Discussion

*M. agalactiae* is the etiological agent of contagious agalactia (CA), a serious disease of sheep and goats reported worldwide and endemic in most Mediterranean countries [32]. The typical symptoms of CA (mastitis, arthritis, keratoconjunctivitis, and occasionally abortion) result in significant economic losses due to a sharp reduction in milk production, to the impaired ability of the host to reproduce, and to the additional expenses associated with therapy, prophylaxis, and diagnosis. Very little is known regarding factors involved in *M. agalactiae* virulence and host interaction. Full genome sequencing of two *M. agalactiae* strains combined to gene ontology analyses [33], [34] revealed that, as in most mycoplasmas, *M. agalactiae* pathogenicity does not relate to primary virulence factors such as cytolysins, invasins, or toxins. Few genes, mostly involved in adhesion, have been identified thus far as related to pathogenicity [35], [36], [37]. Interestingly, lipoproteins modulating both innate and adaptive immune responses are expressed on the *M. agalactiae* membrane [15], [33]. For instance, the membrane expressed P48 lipoprotein has homology to a *M. fermentans* product with a macrophage-stimulatory activity [38]. Also, the Vpma and Avg families of variable lipoproteins provide *M. agalactiae* a mechanism by which it potentially avoids opsonization, phagocytosis, macrophage killing, and antibodies neutralization [39].

Membrane nucleases play a key role for the survival of mycoplasmas into their hosts [40]. Since most mycoplasma species lost essential components of the biochemical machinery in which ribose phosphate, selected amino acids, CO_2_, and NH_3_ are combined in successive reactions to form nucleotides (*de novo* pathway), these microorganisms optimized the use of free bases and nucleosides released from the breakdown of the host nucleic acids by converting them back into nucleotides (salvage pathway, 3, 4]. Under this scenario, membrane nucleases and transport systems for the uptake of nucleotide precursors play not only a crucial functional role in promoting mycoplasmas survival but also contribute to pathogenicity as virulence factors [4], [11], [14], [34], [40], [41]. Indeed, it has been demonstrated that several nucleases are implicated in mycoplasma-mediated host cell cytotoxicity. The *M. pneumoniae* nuclease Mpn133 induces apoptosis-like death of A549 mammalian cells, after binding and internalization [6], [13]. Also, the Ca^2+^ and Mg^2+^ ion-dependent endonucleases produced by *M*. *hyorhinis* and *M*. *penetrans* induce apoptotic changes in epithelial cells, and trigger apoptosis in cultured lymphocytes, respectively [11], [14].

The surface expressed MAG_5040 protein of *M. agalactiae* was found to contain a TNASE_3 thermonuclease domain profile. Recombinant GST-MAG_5040 nuclease activity tested on different nucleic acid substrates showed that MAG_5040 is a sugar-nonspecific exonuclease that preferentially cleaves nucleic acid residues from double and single strand DNA. Slightly smaller exonuclease activity was observed against RNA. Recombinant GST-MAG_5040 also displayed endonuclease activity by nicking closed circular plasmid DNA. Activity of GST-MAG_5040 was optimal in the presence of 20 mM Mg^2+^, and no activity could be detected in the presence of EDTA. These observations are consistent with the identification of binding sites for bivalent ions in the TNASE_3 thermonuclease domain. Mycoplasma nucleases performance is strictly dependent on the presence of divalent cations, and proved optimal in the presence of both magnesium and calcium ions [10], [42], [43]. As an example, the *M. hyopneumoniae* nuclease mhp379 requires only Ca^2+^ [12], while *M. pulmonis*, *M. penetrans*, and *M. hyorhinis* nucleases showed their maximum activity in the presence of both Ca^2+^ and Mg^2+^ ions [8], [16], [42]. Interestingly Ca^2+^ seems to have an inhibitory effect on MAG_5040 activity, as increasing Ca^2+^ concentration results in partial loss of activity already at 2 mM CaCl_2_, and total inhibition at 10 mM. This was already observed in *M. capricolum*, where nuclease is active only in the presence of Mg^2+^ while Ca^2+^ is inhibitory [10]. Nuclease activity of MAG_5040 increased when Na^+^ and K^+^ were added to the reaction at concentrations ranging from 0.1 to 100 mM, while it was dramatically inhibited at 200 mM of any of the two ions. A decrease of such activity with increasing ionic strength has been already observed for other mycoplasma nucleases [10], [12], [42].

Under optimal conditions rGST-MAG_5040 performed best between 30–60°C with maximum activity between 37 and 45°C. This could promote the survival of *M. agalactiae* in poorly thermoregulated external districts of the host, such as the conjunctiva, but also in more controlled environments such as the mammary gland, which under physiological conditions is maintained at 38–40°C temperature. The residual activity of rGST-MAG_5040 at 65°C could be most likely associated with the function of Mg^2+^ in stabilizing the structure of the nuclease, similarly to what observed with Ca^2+^ in analogue experiments conducted on the *M. hyopneumoniae* nuclease mhp379 [12].

In *M. agalactiae* the MAG_5040 gene is located upstream an ABC transporter operon, and this organization is observed in all the mycoplasmas belonging to the *M. hominis* group, and in many other mycoplasma species. The conserved co-localization of the SNase with genes encoding domains associated to transport strongly suggests that MAG_5040 is involved in the import of nucleic acid precursors. Also, homologs of MAG_5030 (P80) can be identified upstream the SNase gene at least in the *M. hominis* group, suggesting its conserved role as solute binding protein. Indeed, MAG_5030 3D modeling and structure prevision designate this protein as belonging to families including solute binding proteins mostly associated with sugar transport. On the contrary, no conserved positions are observed downstream the ABC transporter.

Therefore in a hypothetic model, MAG_5040 could provide nucleotide precursors to the ABC transporter by “stealing” them from the host nucleic acids, with MAG_5030 (P80) acting as solute binding protein. Consequently, MAG_5040 could be a critical pathogenic contributor to *M. agalactiae* persistence by providing essential nucleotide precursors for biosynthesis and replication, while competing with the host for nucleotide pools. The involvement of MAG_5030 and MAG_5040 in an active ABC transport system is supported by the identification of overlapping transcripts between MAG_5030 and MAG_5070 in RT-PCR analyses (data not shown). Moreover a putative transcription promoter is present upstream MAG_5030 while a Rho-independent termination signal can be identified downstream MAG_5080. Notably, MAG_5030, MAG_5040, MAG_5050, MAG_5060, and MAG_5070 are all expressed in cultured *M. agalactiae* PG2^T^ [15].

Sera of sheep and goats naturally infected with *M. agalactiae* collected at different infection times reacted with the recombinant cleaved MAG_5040. On the one hand, the reactivity of sera obtained from outbreaks occurred in distant geographic regions in different years with rMAG_5040, recorded up to 9 months post infection, reinforces the key role of this protein in the interaction with the natural hosts. On the other hand, the establishment of the antigenic properties of MAG_5040 opens new perspectives in the development of both high throughput diagnostic and prophylactic tools for the control of contagious agalactia. If confirmed, the importance of MAG_5040 nuclease in promoting *M. agalactiae* survival and persistence could suggest focusing on this protein as a target for the development of chemotherapics active against nucleotide recycling.

The reactivity of MAG_5040 with rabbit sera raised against selected mycoplasmas suggests the expression of SNase homologs in most of the species examined, including *M. capricolum* and *M. mycoides*. It should be pointed out that a SNase homolog could not be identified by homology search in the genomes of these two latter mycoplasma species. However, our results are in accordance with what experimentally observed by Minion and coworkers, that reported a Mg^2+^ dependent nuclease activity in *M. capricolum* [10].

MAG_5040 is the first antigenic protein with nuclease activity characterized in *M. agalactiae*, potentially involved in pathogenicity and playing an important role in the interaction and survival of this mycoplasma in the host. Further studies, such as functional proteomics assays, might hopefully help to elucidate the interconnected role of MAG_5030, MAG_5040 and of the other components of the putative nucleoside uptake machinery, for the full comprehension of mycoplasmas life cycle, as well as to develop effective tools for the control of mycoplasmosis.

## Supporting Information

Figure S1 **Phyre software results.** Alignment coverage, 3D model, confidence, and percentage of identity of the most similar proteins are shown.
(PDF)

Figure S2 **Expression and purification of rMAG_5040.** MW indicates the molecular weight marker (Precision Plus Protein All Blue, Bio Rad). Lane 1, uninduced *E. coli*. Lane 2, *E. coli* expressing recombinant GST-MAG_5040 after 4 hours induction. Lanes 3 and 4, purified GST-MAG_5040 and its thrombin cleavage products, respectively.
(PDF)

Table S1 **Primers used in this study.**
(PDF)
