# Supplementary material for: Mycoplasma agalactiae MAG_5040 is a Mg2+-Dependent, Sugar-Nonspecific SNase Recognised by the Host Humoral Response during Natural Infection
Source: PLoS One. 2013 Feb 28;8(2):e57775. doi: 10.1371/journal.pone.0057775 (PMC3585158; doi:10.1371/journal.pone.0057775)
Supplement: Figure S1 — Phyre software results. Alignment coverage, 3D model, confidence, and percentage of identity of the most similar proteins are shown. (PDF) [file pone.0057775.s001.pdf]

Phyre2

Email	alberti@uniss.it
Description	p80_____
Date	Tue Oct 30 10:06:31 GMT 2012
Unique Job ID	2d73b8c803f831ec

Detailed template information

#	Template	Alignment Coverage	3D Model	Confidence	% i.d.	Template Information
1	d1eu8a_	Alignment	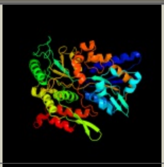	99.6	16	**Fold:**Periplasmic binding protein-like II **Superfamily:**Periplasmic binding protein-like II **Family:**Phosphate binding protein-like
2	c3mp6A_	Alignment	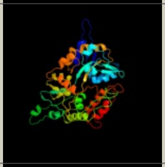	99.5	17	**PDB header:**histone binding protein **Chain:** A: **PDB Molecule:**maltose-binding periplasmic protein, linker, saga- **PDBTitle:** complex structure of sgf29 and dimethylated h3k4
3	c3qufB_	Alignment	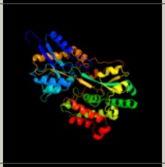	99.4	11	**PDB header:**transport protein **Chain:** B: **PDB Molecule:**extracellular solute-binding protein, family 1; **PDBTitle:** the structure of a family 1 extracellular solute-binding protein from2 bifidobacterium longum subsp. infantis
4	c1y4cA_	Alignment	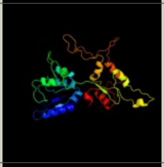	99.4	12	**PDB header:**de novo protein **Chain:** A: **PDB Molecule:**maltose binding protein fused with designed **PDBTitle:** designed helical protein fusion mbp
5	d1elja_	Alignment	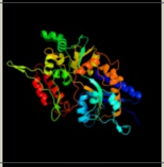	99.4	14	**Fold:**Periplasmic binding protein-like II **Superfamily:**Periplasmic binding protein-like II **Family:**Phosphate binding protein-like
6	c4aq4A_	Alignment	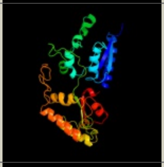	99.4	15	**PDB header:**diester-binding protein **Chain:** A: **PDB Molecule:**sn-glycerol-3-phosphate-binding periplasmic protein ugpb; **PDBTitle:** substrate bound sn-glycerol-3-phosphate binding periplasmic protein2 ugpb from escherichia coli
7	c4h1gA_	Alignment	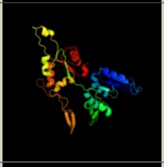	99.4	11	**PDB header:**motor protein **Chain:** A: **PDB Molecule:**maltose binding protein-cakar3 motor domain fusion protein; **PDBTitle:** structure of candida albicans kar3 motor domain fused to maltose-2 binding protein
8	c2nvuB_	Alignment	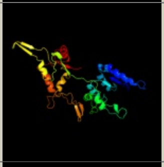	99.4	12	**PDB header:**protein turnover, ligase **Chain:** B: **PDB Molecule:**maltose binding protein/nedd8-activating enzyme **PDBTitle:** structure of appbp1-uba3~nedd8-nedd8-mgatp-ubc12(c111a), a2 trapped ubiquitin-like protein activation complex
9	c3osqA_	Alignment	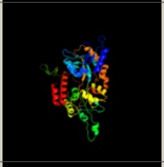	99.4	20	**PDB header:**fluorescent protein, transport protein **Chain:** A: **PDB Molecule:**maltose-binding periplasmic protein, green fluorescent **PDBTitle:** maltose-bound maltose sensor engineered by insertion of circularly2 permuted green fluorescent protein into e. coli maltose binding3 protein at position 175
10	c3ob4A_	Alignment	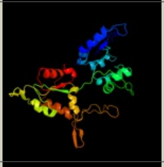	99.4	11	**PDB header:**allergen **Chain:** A: **PDB Molecule:**maltose abc transporter periplasmic protein, arah 2; **PDBTitle:** mbp-fusion protein of the major peanut allergen ara h 2
11	c3f5fA_	Alignment	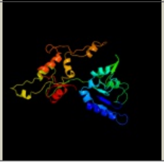	99.3	12	**PDB header:**transport, transferase **Chain:** A: **PDB Molecule:**maltose-binding periplasmic protein, heparan **PDBTitle:** crystal structure of heparan sulfate 2-o-sulfotransferase2 from gallus gallus as a maltose binding protein fusion.

12	c4g68C_	Alignment	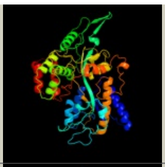	99.3	16	**PDB header:**transport protein<br>**Chain:** C: **PDB Molecule:**abc transporter;<br>**PDBTitle:** biochemical and structural insights into xylan utilization by the2 thermophilic bacteriumcaldanaerobius polysaccharolyticus
13	c3c4mA_	Alignment	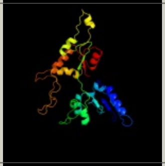	99.3	12	**PDB header:**membrane protein<br>**Chain:** A: **PDB Molecule:**fusion protein of maltose-binding periplasmic protein and<br>**PDBTitle:** structure of human parathyroid hormone in complex with the2 extracellular domain of its g-protein-coupled receptor (pth1r)
14	c3dm0A_	Alignment	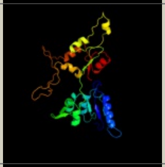	99.3	12	**PDB header:**sugar binding protein,signaling protein<br>**Chain:** A: **PDB Molecule:**maltose-binding periplasmic protein fused with<br>**PDBTitle:** maltose binding protein fusion with rack1 from a. thaliana
15	c4gqoC_	Alignment	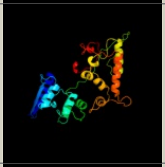	99.3	15	**PDB header:**unknown function<br>**Chain:** C: **PDB Molecule:**lmo0859 protein;<br>**PDBTitle:** 2.1 angstrom resolution crystal structure of uncharacterized protein2 lmo0859 from listeria monocytogenes egd-e
16	c1hsjA_	Alignment	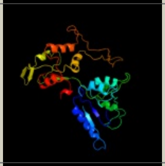	99.3	12	**PDB header:**transcription/sugar binding protein<br>**Chain:** A: **PDB Molecule:**fusion protein consisting of staphylococcus<br>**PDBTitle:** sarr mbp fusion structure
17	c3o3uN_	Alignment	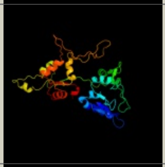	99.3	12	**PDB header:**transport protein, signaling protein<br>**Chain:** N: **PDB Molecule:**maltose-binding periplasmic protein, advanced glycosylation<br>**PDBTitle:** crystal structure of human receptor for advanced glycation endproducts2 (rage)
18	c2z8fB_	Alignment	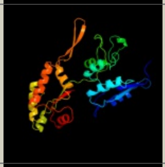	99.3	13	**PDB header:**sugar binding protein<br>**Chain:** B: **PDB Molecule:**galacto-n-biose/lacto-n-biose i transporter substrate-<br>**PDBTitle:** the galacto-n-biose-/lacto-n-biose i-binding protein (gl-bp) of the2 abc transporter from bifidobacterium longum in complex with lacto-n-3 tetraose
19	c3k02A_	Alignment	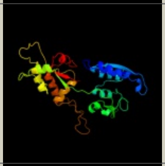	99.3	15	**PDB header:**transport protein<br>**Chain:** A: **PDB Molecule:**acarbose/maltose binding protein gach;<br>**PDBTitle:** crystal structures of the gach receptor of streptomyces glaucescens2 gla.o in the unliganded form and in complex with acarbose and an3 acarbose homolog. comparison with acarbose-loaded maltose binding4 protein of salmonella typhimurium.
20	c2vgqA_	Alignment	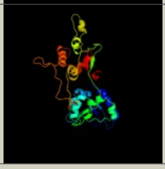	99.3	12	**PDB header:**immune system/transport<br>**Chain:** A: **PDB Molecule:**maltose-binding periplasmic protein,<br>**PDBTitle:** crystal structure of human ips-1 card
21	c3py7A_	Alignment	not modelled	99.3	12	**PDB header:**viral protein<br>**Chain:** A: **PDB Molecule:**maltose-binding periplasmic protein,paxillin ld1,protein e6<br>**PDBTitle:** crystal structure of full-length bovine papillomavirus oncoprotein e62 in complex with ld1 motif of paxillin at 2.3a resolution
22	c2gh9A_	Alignment	not modelled	99.3	16	**PDB header:**sugar binding protein<br>**Chain:** A: **PDB Molecule:**maltose/maltodextrin-binding protein;<br>**PDBTitle:** thermus thermophilus maltotriose binding protein bound with2 maltotriose
23	c3oaiB_	Alignment	not modelled	99.3	11	**PDB header:**membrane protein, cell adhesion<br>**Chain:** B: **PDB Molecule:**maltose-binding periplasmic protein, myelin protein p0;<br>**PDBTitle:** crystal structure of the extra-cellular domain of human myelin protein2 zero
24	c4b3nA_	Alignment	not modelled	99.3	12	**PDB header:**sugar binding protein/ligase<br>**Chain:** A: **PDB Molecule:**maltose-binding periplasmic protein, tripartite<br>**PDBTitle:** crystal structure of rhesus trim5alpha pry/spry domain
25	c3d4cA_	Alignment	not modelled	99.3	11	**PDB header:**cell adhesion<br>**Chain:** A: **PDB Molecule:**maltose-binding periplasmic protein, linker, zona pellucida<br>**PDBTitle:** zp-n domain of mammalian sperm receptor zp3 (crystal form i)
26	c4dxbB_	Alignment	not modelled	99.3	12	**PDB header:**sugar binding protein, hydrolase<br>**Chain:** B: **PDB Molecule:**maltose-binding periplasmic protein, beta-lactamase tem<br>**PDBTitle:** 2.29a structure of the engineered mbp tem-1 fusion protein rg13 in2 complex with zinc, p1 space group
27	c1r6zA_	Alignment	not modelled	99.3	11	**PDB header:**gene regulation<br>**Chain:** A: **PDB Molecule:**chimera of maltose-binding periplasmic protein and

		Alignment				**PDBTitle:** the crystal structure of the argonaute2 paz domain (as a mbp fusion)
28	c3i3vC_	Alignment	not modelled	99.3	13	**PDB header:**transport protein<br>**Chain:** C: **PDB Molecule:**probable secreted solute-binding lipoprotein;<br>**PDBTitle:** crystal structure of probable secreted solute-binding2 lipoprotein from streptomyces coelicolor
29	c3oo6A_	Alignment	not modelled	99.3	15	**PDB header:**sugar binding protein<br>**Chain:** A: **PDB Molecule:**abc transporter binding protein acbh;<br>**PDBTitle:** crystal structures and biochemical characterization of the bacterial2 solute receptor acbh reveal an unprecedented exclusive substrate3 preference for b-d-galactopyranose
30	c3ehuA_	Alignment	not modelled	99.3	12	**PDB header:**membrane protein<br>**Chain:** A: **PDB Molecule:**fusion protein of crfr1 extracellular domain and mbp;<br>**PDBTitle:** crystal structure of the extracellular domain of human corticotropin2 releasing factor receptor type 1 (crfr1) in complex with crf
31	c3csgA_	Alignment	not modelled	99.2	12	**PDB header:**de novo protein, sugar binding protein<br>**Chain:** A: **PDB Molecule:**maltose-binding protein monobody ys1 fusion;<br>**PDBTitle:** crystal structure of monobody ys1(mbp-74)/maltose binding2 protein fusion complex
32	c3iouB_	Alignment	not modelled	99.2	16	**PDB header:**signaling protein<br>**Chain:** B: **PDB Molecule:**maltose-binding protein, huntingtin fusion<br>**PDBTitle:** huntingtin amino-terminal region with 17 gln residues -2 crystal c94
33	c3uorB_	Alignment	not modelled	99.2	9	**PDB header:**sugar binding protein<br>**Chain:** B: **PDB Molecule:**abc transporter sugar binding protein;<br>**PDBTitle:** the structure of the sugar-binding protein male from the phytopathogen2 xanthomonas citri
34	c3osrA_	Alignment	not modelled	99.2	12	**PDB header:**fluorescent protein, transport protein<br>**Chain:** A: **PDB Molecule:**maltose-binding periplasmic protein, green fluorescent<br>**PDBTitle:** maltose-bound maltose sensor engineered by insertion of circularly2 permuted green fluorescent protein into e. coli maltose binding3 protein at position 311
35	c3h4zC_	Alignment	not modelled	99.2	11	**PDB header:**allergen<br>**Chain:** C: **PDB Molecule:**maltose-binding periplasmic protein fused with allergen<br>**PDBTitle:** crystal structure of an mbp-der p 7 fusion protein
36	c2fncA_	Alignment	not modelled	99.2	11	**PDB header:**sugar binding protein<br>**Chain:** A: **PDB Molecule:**maltose abc transporter, periplasmic maltose-binding<br>**PDBTitle:** thermotoga maritima maltotriose binding protein bound with2 maltotriose.
37	c4exkA_	Alignment	not modelled	99.1	11	**PDB header:**transport protein<br>**Chain:** A: **PDB Molecule:**maltose-binding periplasmic protein, uncharacterized<br>**PDBTitle:** a chimera protein containing mbp fused to the c-terminal domain of the2 uncharacterized protein stm14_2015 from salmonella enterica
38	c1mg1A_	Alignment	not modelled	99.1	11	**PDB header:**viral protein<br>**Chain:** A: **PDB Molecule:**protein (htlv-1 gp21 ectodomain/maltose-binding protein<br>**PDBTitle:** htlv-1 gp21 ectodomain/maltose-binding protein chimera
39	c2uvgA_	Alignment	not modelled	98.9	10	**PDB header:**sugar-binding protein<br>**Chain:** A: **PDB Molecule:**abc type periplasmic sugar-binding protein;<br>**PDBTitle:** structure of a periplasmic oligogalacturonide binding2 protein from yersinia enterocolitica
40	c2w7yA_	Alignment	not modelled	98.9	13	**PDB header:**sugar-binding protein<br>**Chain:** A: **PDB Molecule:**probable sugar abc transporter, sugar-binding<br>**PDBTitle:** structure of a streptococcus pneumoniae solute-binding2 protein in complex with the blood group a-trisaccharide.
41	c1svxB_	Alignment	not modelled	98.9	11	**PDB header:**de novo protein/sugar binding protein<br>**Chain:** B: **PDB Molecule:**maltose-binding periplasmic protein;<br>**PDBTitle:** crystal structure of a designed selected ankyrin repeat2 protein in complex with the maltose binding protein
42	c4g68A_	Alignment	not modelled	98.8	16	**PDB header:**transport protein<br>**Chain:** A: **PDB Molecule:**abc transporter;<br>**PDBTitle:** biochemical and structural insights into xylan utilization by the2 thermophilic bacteriumcaldanaerobius polysaccharolyticus
43	c2zykA_	Alignment	not modelled	98.7	9	**PDB header:**sugar binding protein<br>**Chain:** A: **PDB Molecule:**solute-binding protein;<br>**PDBTitle:** crystal structure of cyclo/maltodextrin-binding protein2 complexed with gamma-cyclodextrin
44	d1laxa_	Alignment	not modelled	98.7	12	**Fold:**Periplasmic binding protein-like II<br>**Superfamily:**Periplasmic binding protein-like II<br>**Family:**Phosphate binding protein-like
45	d1j1na_	Alignment	not modelled	98.6	10	**Fold:**Periplasmic binding protein-like II<br>**Superfamily:**Periplasmic binding protein-like II<br>**Family:**Phosphate binding protein-like
46	d1y3na1	Alignment	not modelled	98.6	9	**Fold:**Periplasmic binding protein-like II<br>**Superfamily:**Periplasmic binding protein-like II<br>**Family:**Phosphate binding protein-like
47	c1mh3A_	Alignment	not modelled	98.4	11	**PDB header:**sugar binding, dna binding protein<br>**Chain:** A: **PDB Molecule:**maltose binding-a1 homeodomain protein chimera;<br>**PDBTitle:** maltose binding-a1 homeodomain protein chimera, crystal2 form i
48	c2i58B_	Alignment	not modelled	98.3	13	**PDB header:**sugar binding protein<br>**Chain:** B: **PDB Molecule:**sugar abc transporter, sugar-binding protein;<br>**PDBTitle:** crystal structure of rafe from streptococcus pneumoniae complexed with2 raffinose
49	d3thia_	Alignment	not modelled	98.3	8	**Fold:**Periplasmic binding protein-like II<br>**Superfamily:**Periplasmic binding protein-like II<br>**Family:**Phosphate binding protein-like

50	c2xd3A_	Alignment	not modelled	98.2	15	**PDB header:**sugar binding protein<br>**Chain:** A: **PDB Molecule:**maltose/maltodextrin-binding protein;<br>**PDBTitle:** the crystal structure of malx from streptococcus pneumoniae2 in complex with maltopentaose.
51	c2b3fD_	Alignment	not modelled	98.1	12	**PDB header:**sugar binding protein<br>**Chain:** D: **PDB Molecule:**glucose-binding protein;<br>**PDBTitle:** thermus thermophilus glucose/galactose binding protein2 bound with galactose
52	c3ombA_	Alignment	not modelled	98.1	10	**PDB header:**transport protein<br>**Chain:** A: **PDB Molecule:**extracellular solute-binding protein, family 1;<br>**PDBTitle:** crystal structure of extracellular solute-binding protein from2 bifidobacterium longum subsp. infantis
53	d1pota_	Alignment	not modelled	97.9	15	**Fold:**Periplasmic binding protein-like II<br>**Superfamily:**Periplasmic binding protein-like II<br>**Family:**Phosphate binding protein-like
54	c3ttkA_	Alignment	not modelled	97.8	11	**PDB header:**transport protein<br>**Chain:** A: **PDB Molecule:**polyamine transport protein;<br>**PDBTitle:** crystal structure of apo-spud
55	c1ursA_	Alignment	not modelled	97.8	13	**PDB header:**maltose-binding protein<br>**Chain:** A: **PDB Molecule:**maltose-binding protein;<br>**PDBTitle:** x-ray structures of the maltose-maltodextrin binding2 protein of the thermoacidophilic bacterium alicyclobacillus3 acidocaldarius
56	d1ursa_	Alignment	not modelled	97.8	13	**Fold:**Periplasmic binding protein-like II<br>**Superfamily:**Periplasmic binding protein-like II<br>**Family:**Phosphate binding protein-like
57	c3ttlB_	Alignment	not modelled	97.6	13	**PDB header:**transport protein<br>**Chain:** B: **PDB Molecule:**polyamine transport protein;<br>**PDBTitle:** crystal structure of apo-spue
58	c3c9hB_	Alignment	not modelled	97.5	21	**PDB header:**transport protein<br>**Chain:** B: **PDB Molecule:**abc transporter, substrate binding protein;<br>**PDBTitle:** crystal structure of the substrate binding protein of the abc2 transporter from agrobacterium tumefaciens
59	d1xvxa_	Alignment	not modelled	97.5	15	**Fold:**Periplasmic binding protein-like II<br>**Superfamily:**Periplasmic binding protein-like II<br>**Family:**Phosphate binding protein-like
60	c4eqbA_	Alignment	not modelled	97.3	14	**PDB header:**transport protein<br>**Chain:** A: **PDB Molecule:**spermidine/putrescine abc superfamily atp binding cassette<br>**PDBTitle:** 1.5 angstrom crystal structure of spermidine/putrescine abc2 transporter substrate-binding protein from streptococcus pneumoniae3 strain canada mdr_19a in complex with calcium and hepes.
61	d2onsa1	Alignment	not modelled	97.1	9	**Fold:**Periplasmic binding protein-like II<br>**Superfamily:**Periplasmic binding protein-like II<br>**Family:**Phosphate binding protein-like
62	c2v84A_	Alignment	not modelled	97.1	14	**PDB header:**transport protein<br>**Chain:** A: **PDB Molecule:**spermidine/putrescine abc transporter, periplasmic<br>**PDBTitle:** crystal structure of the tp0655 (tppotd) lipoprotein of2 treponema pallidum
63	c4edpA_	Alignment	not modelled	97.0	15	**PDB header:**transport protein<br>**Chain:** A: **PDB Molecule:**abc transporter, substrate-binding protein;<br>**PDBTitle:** 1.85 angstrom resolution crystal structure of an abc transporter from2 clostridium perfringens atcc 13124
64	c2pt1A_	Alignment	not modelled	97.0	12	**PDB header:**metal transport<br>**Chain:** A: **PDB Molecule:**iron transport protein;<br>**PDBTitle:** futa1 synechocystis pcc 6803
65	c4gl0A_	Alignment	not modelled	96.9	10	**PDB header:**transport protein<br>**Chain:** A: **PDB Molecule:**lmo0810 protein;<br>**PDBTitle:** putative spermidine/putrescine abc transporter from listeria2 monocytogenes
66	c3pu5A_	Alignment	not modelled	96.8	9	**PDB header:**transport protein<br>**Chain:** A: **PDB Molecule:**extracellular solute-binding protein;<br>**PDBTitle:** the crystal structure of a putative extracellular solute-binding2 protein from bordetella parapertussis
67	d1xvya_	Alignment	not modelled	96.8	16	**Fold:**Periplasmic binding protein-like II<br>**Superfamily:**Periplasmic binding protein-like II<br>**Family:**Phosphate binding protein-like
68	d1y4ta_	Alignment	not modelled	96.7	11	**Fold:**Periplasmic binding protein-like II<br>**Superfamily:**Periplasmic binding protein-like II<br>**Family:**Phosphate binding protein-like
69	c3rpwA_	Alignment	not modelled	96.5	13	**PDB header:**transport protein<br>**Chain:** A: **PDB Molecule:**abc transporter;<br>**PDBTitle:** the crystal structure of an abc transporter from rhodopseudomonas2 palustris cga009
70	d1q35a_	Alignment	not modelled	96.4	14	**Fold:**Periplasmic binding protein-like II<br>**Superfamily:**Periplasmic binding protein-like II<br>**Family:**Phosphate binding protein-like
71	d1xc1a_	Alignment	not modelled	96.3	8	**Fold:**Periplasmic binding protein-like II<br>**Superfamily:**Periplasmic binding protein-like II<br>**Family:**Phosphate binding protein-like
72	d1nnfa_	Alignment	not modelled	96.3	10	**Fold:**Periplasmic binding protein-like II<br>**Superfamily:**Periplasmic binding protein-like II<br>**Family:**Phosphate binding protein-like
73	c2qryD_	Alignment	not modelled	95.7	12	**PDB header:**transport protein<br>**Chain:** D: **PDB Molecule:**thiamine-binding periplasmic protein;<br>**PDBTitle:** periplasmic thiamin binding protein
74	d1a99a_	Alignment	not modelled	95.6	9	**Fold:**Periplasmic binding protein-like II<br>**Superfamily:**Periplasmic binding protein-like II

						**Family:**Phosphate binding protein-like
75	c2vozA_	Alignment	not modelled	95.6	11	**PDB header:**metal-binding protein<br>**Chain:** A: **PDB Molecule:**periplasmic iron-binding protein;<br>**PDBTitle:** apo futa2 from synechocystis pcc6803
76	c3cfzA_	Alignment	not modelled	95.3	16	**PDB header:**transport protein<br>**Chain:** A: **PDB Molecule:**upf0100 protein mj1186;<br>**PDBTitle:** crystal structure of m. jannaschii periplasmic binding2 protein moda/wtpa with bound tungstate
77	c3k6wA_	Alignment	not modelled	93.7	14	**PDB header:**transport protein<br>**Chain:** A: **PDB Molecule:**solute-binding protein ma_0280;<br>**PDBTitle:** apo and ligand bound structures of moda from the archaeon2 methanosarcina acetivorans
78	c3cg3A_	Alignment	not modelled	93.5	14	**PDB header:**transport protein<br>**Chain:** A: **PDB Molecule:**upf0100 protein ph0151;<br>**PDBTitle:** crystal structure of p. horikoshii periplasmic binding2 protein moda/wtpa with bound tungstate
79	d1y9ua_	Alignment	not modelled	92.7	14	**Fold:**Periplasmic binding protein-like II<br>**Superfamily:**Periplasmic binding protein-like II<br>**Family:**Phosphate binding protein-like
80	c3cg1A_	Alignment	not modelled	91.5	13	**PDB header:**transport protein<br>**Chain:** A: **PDB Molecule:**upf0100 protein pf0080;<br>**PDBTitle:** crystal structure of p. furiosus periplasmic binding protein2 moda/wtpa with bound tungstate
81	d1atga_	Alignment	not modelled	83.1	22	**Fold:**Periplasmic binding protein-like II<br>**Superfamily:**Periplasmic binding protein-like II<br>**Family:**Phosphate binding protein-like
82	c3cfxA_	Alignment	not modelled	79.7	16	**PDB header:**transport protein<br>**Chain:** A: **PDB Molecule:**upf0100 protein ma_0280;<br>**PDBTitle:** crystal structure of m. acetivorans periplasmic binding protein2 moda/wtpa with bound tungstate
83	c1jrjA_	Alignment	not modelled	79.5	19	**PDB header:**hormone/growth factor<br>**Chain:** A: **PDB Molecule:**exendin-4;<br>**PDBTitle:** solution structure of exendin-4 in 30-vol% trifluoroethanol
84	c1d0rA_	Alignment	not modelled	75.8	31	**PDB header:**hormone/growth factor<br>**Chain:** A: **PDB Molecule:**glucagon-like peptide-1-(7-36)-amide;<br>**PDBTitle:** solution structure of glucagon-like peptide-1-(7-36)-amide2 in trifluoroethanol/water
85	d1twya_	Alignment	not modelled	75.0	23	**Fold:**Periplasmic binding protein-like II<br>**Superfamily:**Periplasmic binding protein-like II<br>**Family:**Phosphate binding protein-like
86	c1twyG_	Alignment	not modelled	73.5	23	**PDB header:**structural genomics, unknown function<br>**Chain:** G: **PDB Molecule:**abc transporter, periplasmic substrate-binding protein;<br>**PDBTitle:** crystal structure of an abc-type phosphate transport receptor from2 vibrio cholerae
87	c2l64A_	Alignment	not modelled	68.1	26	**PDB header:**hormone<br>**Chain:** A: **PDB Molecule:**glucagon-like peptide 2;<br>**PDBTitle:** nmr solution structure of glp-2 in dhpc micelles
88	c3lr1A_	Alignment	not modelled	64.8	8	**PDB header:**transport protein<br>**Chain:** A: **PDB Molecule:**tungstate abc transporter, periplasmic tungstate-<br>**PDBTitle:** the crystal structure of the tungstate abc transporter from2 geobacter sulfurreducens
89	c4gd5B_	Alignment	not modelled	59.5	21	**PDB header:**transport protein<br>**Chain:** B: **PDB Molecule:**phosphate abc transporter, phosphate-binding protein;<br>**PDBTitle:** x-ray crystal structure of a putative phosphate abc transporter2 substrate-binding protein with bound phosphate from clostridium3 perfringens
90	c1t5qA_	Alignment	not modelled	51.0	33	**PDB header:**hormone/growth factor<br>**Chain:** A: **PDB Molecule:**gastric inhibitory polypeptide;<br>**PDBTitle:** solution structure of gip(1-30)amide in tfe/water
91	c3c5tB_	Alignment	not modelled	50.8	20	**PDB header:**signaling protein/signaling protein<br>**Chain:** B: **PDB Molecule:**exendin-4;<br>**PDBTitle:** crystal structure of the ligand-bound glucagon-like peptide-1 receptor2 extracellular domain
92	c1bh0A_	Alignment	not modelled	42.9	27	**PDB header:**synthetic hormone<br>**Chain:** A: **PDB Molecule:**glucagon;<br>**PDBTitle:** structure of a glucagon analog
93	c3f7cA_	Alignment	not modelled	40.9	15	**PDB header:**unknown function<br>**Chain:** A: **PDB Molecule:**protein of unknown function (duf416);<br>**PDBTitle:** crystal structure of a duf416 family protein (maqu_0942) from2 marinobacter aquaeolei vt8 at 2.00 a resolution
94	c2obuA_	Alignment	not modelled	38.7	33	**PDB header:**hormone/growth factor<br>**Chain:** A: **PDB Molecule:**gastric inhibitory polypeptide;<br>**PDBTitle:** solution structure of gip in tfe/water
95	c2q9rA_	Alignment	not modelled	38.6	13	**PDB header:**unknown function<br>**Chain:** A: **PDB Molecule:**protein of unknown function;<br>**PDBTitle:** crystal structure of a duf416 family protein (sbal_3149) from2 shewanella baltica os155 at 1.91 a resolution
96	c3kn3C_	Alignment	not modelled	36.7	17	**PDB header:**transcription<br>**Chain:** C: **PDB Molecule:**putative periplasmic protein;<br>**PDBTitle:** crystal structure of lysr substrate binding domain (25-263) of2 putative periplasmic protein from wolinella succinogenes
97	c3muqB_	Alignment	not modelled	36.7	17	**PDB header:**structural genomics, unknown function<br>**Chain:** B: **PDB Molecule:**uncharacterized conserved protein;<br>**PDBTitle:** the crystal structure of a conserved functionally unknown protein from2 vibrio parahaemolyticus rimd 2210633
98	c3fj7A_	Alignment	not modelled	36.6	18	**PDB header:**protein binding<br>**Chain:** A: **PDB Molecule:**major antigenic peptide peb3;<br>**PDBTitle:** crystal structure of l-phospholactate bound peb3
99	c2hdlA_	Alignment	not modelled	35.5	30	**PDB header:**cytokine<br>**Chain:** A: **PDB Molecule:**small inducible cytokine b14;<br>**PDBTitle:** solution structure of brak/cxcl14
