# Supplementary figures and images for: Mycoplasma agalactiae MAG_5040 is a Mg2+-Dependent, Sugar-Nonspecific SNase Recognised by the Host Humoral Response during Natural Infection
Source: PLoS One. 2013 Feb 28;8(2):e57775. doi: 10.1371/journal.pone.0057775 (PMC3585158; doi:10.1371/journal.pone.0057775)

**MW**

**1**

**2**

**3**

**4**

**70 kDa**

**50 kDa**

**30 kDa**

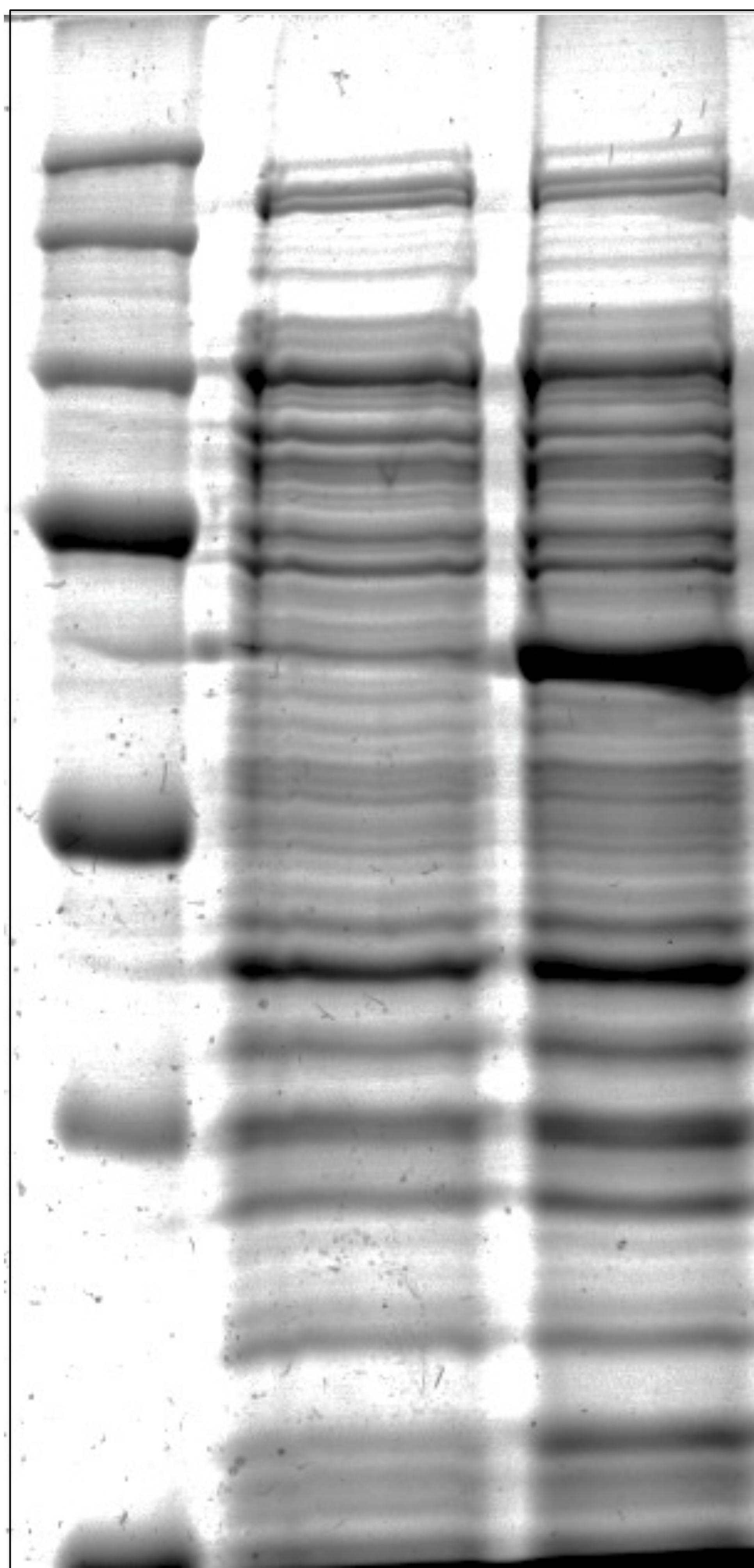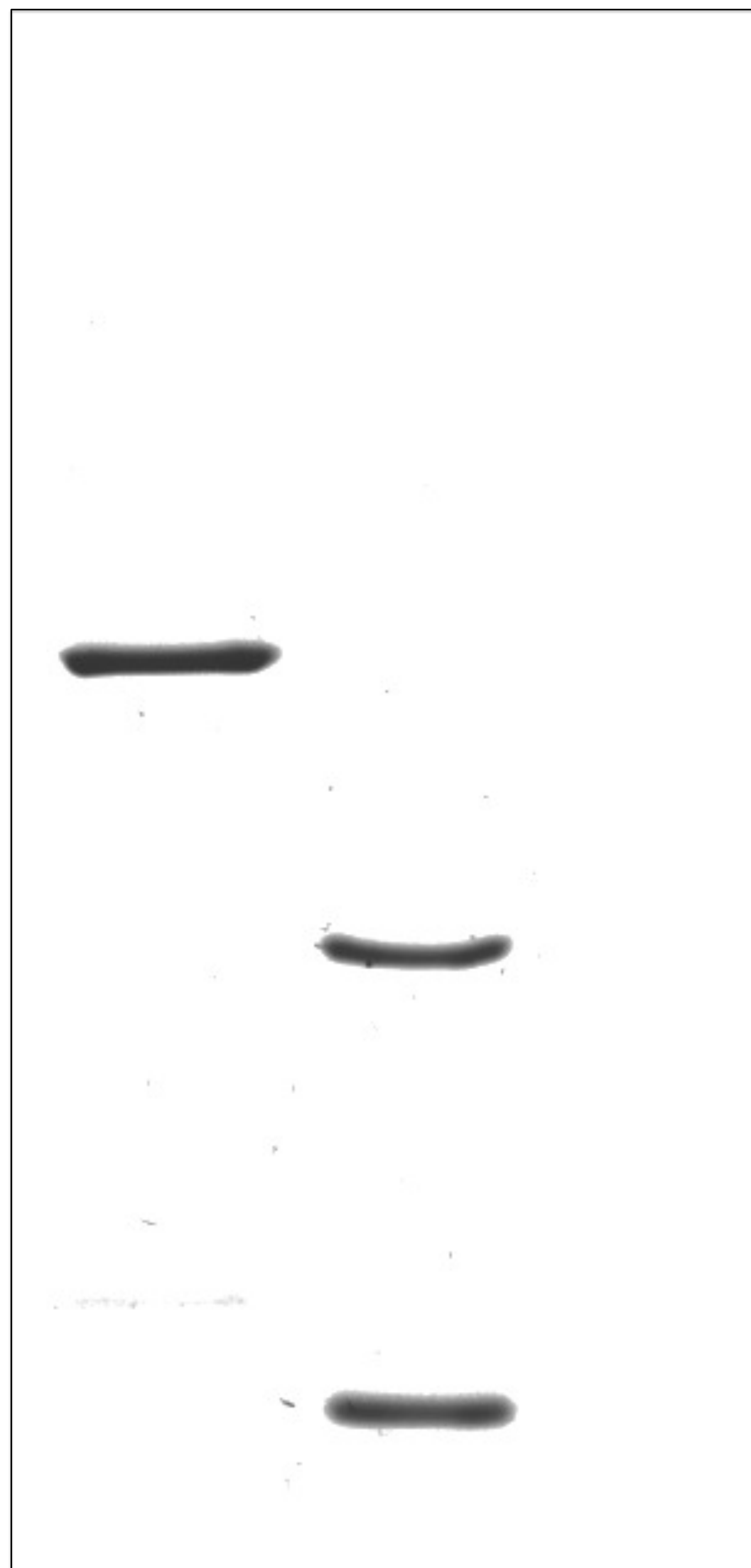

Supplement: Figure S2 — Expression and purification of rMAG_5040. MW indicates the molecular weight marker (Precision Plus Protein All Blue, Bio Rad). Lane 1, uninduced E. coli. Lane 2, E. coli expressing recombinant GST-MAG_5040 after 4 hours induction. Lanes 3 and 4, purified GST-MAG_5040 and its thrombin cleavage products, respectively. (PDF) [file pone.0057775.s002.pdf]
