# Supplementary material for: Mycoplasma agalactiae MAG_5040 is a Mg2+-Dependent, Sugar-Nonspecific SNase Recognised by the Host Humoral Response during Natural Infection
Source: PLoS One. 2013 Feb 28;8(2):e57775. doi: 10.1371/journal.pone.0057775 (PMC3585158; doi:10.1371/journal.pone.0057775)
Supplement: Table S1 — Primers used in this study. (PDF) [file pone.0057775.s003.pdf]

TABLE S1. Primers used in this work.

PRIMER NAME	SEQUENCE (5'→3')	USE
MAG_5040/BamHI/F	CGCGGATCCGATAAAGGCACAATTAAAGC	Cohesive ends for pGEX2T
MAG_5040/EcoRI/R	CCCGAATTCTTATTTGTTTTTGTATGAATCATTGATC	Cohesive ends for pGEX2T
MAG_5040/MUT1/F	GTTGAATTAGTTGATGGCTGGAATAGAAAATTAAGCATAAAA	Site directed mutagenesis
MAG_5040/MUT1/R	TTTTATGCTTAATTTTCTATTCCAGCCATCAACTAATTCAAC	Site directed mutagenesis
MAG_5040/MUT2/F	CGTTTGAGTTTAAAAATATAGACTGGAATAAACTAAAAGCTAAATATTTTG	Site directed mutagenesis
MAG_5040/MUT2/R	CAAAATATTTAGCTTTTAGTTTATTCCAGTCTATATTTTTAAACTCAAACG	Site directed mutagenesis
MAG_5040/MUT3/F	CTGAAATTGTAGAATGGAGCGATGGTGATACAC	Site directed mutagenesis
MAG_5040/MUT3/R	GTGTATCACCATCGCTCCATTCTACAATTTCAG	Site directed mutagenesis
MAG_5040/MUT4/F	GCTTTTGGAAGATGGGTGGGTGATGTGTTC	Site directed mutagenesis
MAG_5040/MUT4/R	GAACACATCACCCACCCATCTTCCAAAAGC	Site directed mutagenesis
MAG_5040/MUT5/F	CAAAAACCCTCTTTACTGGGAACACTATGCGCTTTTAC	Site directed mutagenesis
MAG_5040/MUT5/R	GTAAAAGCGCATAGTGTTCCCAGTAAAGAGGGTTTTTG	Site directed mutagenesis
MAG_5040/MUT6/F	CAAAACTTTTGCTTTGCCATACTGGATTCAATCAAATATTTATAGAATG	Site directed mutagenesis
MAG_5040/MUT6/R	CATTCTATAAATATTTGATTGAATCCAGTATGGCAAAGCAAAAGTTTTG	Site directed mutagenesis
MAG_5040/MUT7/F	GAATGAAACCTAATGGTAAATGGACTTTATTAGATAAAAAATCTG	Site directed mutagenesis
MAG_5040/MUT7/R	CAGATTTTTTATCTAATAAAGTCCATTTACCATTAGGTTTCATT	Site directed mutagenesis
UvrC/end/F	GCATCAGTTGAAGAATTAGC	RT-PCR
